# A small molecule inhibitor of PAI-1 protects against doxorubicin-induced cellular senescence

**DOI:** 10.18632/oncotarget.12494

**Published:** 2016-10-06

**Authors:** Asish K. Ghosh, Rahul Rai, Kitae E. Park, Mesut Eren, Toshio Miyata, Lisa D. Wilsbacher, Douglas E. Vaughan

**Affiliations:** ^1^ Feinberg Cardiovascular Research Institute, Northwestern University Feinberg School of Medicine, Chicago, Illinois, USA; ^2^ United Centers for Advanced Research and Translational Medicine, Tohoku University, Miyagi, Japan

**Keywords:** cellular senescence, doxorubicin, cardiomyocytes, fibroblasts, endothelial cells, Gerotarget

## Abstract

Doxorubicin, an anthracycline antibiotic, is a commonly used anticancer drug. In spite of its widespread usage, its therapeutic effect is limited by its cardiotoxicity. On the cellular level, Doxorubicin-induced cardiotoxicity manifests as stress induced premature senescence. Previously, we demonstrated that plasminogen activator inhibitor-1 (PAI-1), a potent inhibitor of serine proteases, is an important biomarker and regulator of cellular senescence and aging. Here, we tested the hypothesis that pharmacological inhibition of cellular PAI-1 protects against stress- and aging-induced cellular senescence and delineated the molecular basis of protective action of PAI-1 inhibition. Results show that TM5441, a potent small molecule inhibitor of PAI-1, effectively prevents Doxorubicin-induced senescence in cardiomyocytes, fibroblasts and endothelial cells. TM5441 exerts its inhibitory effect on Doxorubicin-induced cellular senescence by decreasing reactive oxygen species generation, induction of antioxidants like catalase and suppression of stress-induced senescence cadre p53, p21, p16, PAI-1 and IGFBP3. Importantly, TM5441 also reduces replicative senescence of fibroblasts. Together these results for the first time demonstrate the efficacy of PAI-1 inhibitor in prevention of Doxorubicin-induced and replicative senescence in normal cells. Thus PAI-1 inhibitor may form an important adjuvant component of chemotherapy regimens, limiting not only Doxorubicin-induced cardiac senescence but also ameliorating the prothrombotic profile.

## INTRODUCTION

Senescence is a cellular process by which cells stop proliferation irreversibly and undergo morphological changes due to expression of high levels of cell cycle inhibitors and sub-set of genes whose products are involved in senescence [1, 2]. In contrast, due to contact inhibition cultured normal cells in confluency undergo reversible growth arrest. Geroconversion, a cellular and molecular process of conversion of reversible growth arrest to irreversible growth arrest, leads to senescence [3, 4]. Although senescent cells are not proliferating or quiescent, they are metabolically active and synthesize different pools of cytokines, growth factors and other regulatory enzymes including IL-6, IL8, IGFBP3, TNFα, PAI-1, TGF-β etc. and collectively known as senescence-associated secretory phenotype (SASP) or senescence messaging secretome (SMS) [1, 2]. The SASP plays a vital role in modulation of gene expression pattern that is different from proliferating and quiescent cells and contributes to stimulation and maintenance of senescence features [1, 5]. Through progressive cellular division, the telomere length ultimately shortens leading to cellular growth arrest and replicative senescence. However, cells may undergo senescence prematurely due to exposure to a variety of stresses or insults which is known as stress-activated or induced senescence [1, 2, 5, 6]. The consequences of senescence in biological processes are both beneficial and detrimental because while senescence of highly mitotic preneoplastic cells is desirable, premature senescence of normal cells is associated with unwanted accelerated aging processes and adverse side-effects in patients treated with oxidative stressors like Doxorubicin. Senescence can also be induced in cultured cells using a wide variety of senescence-inducing stimuli including culture shock stress, high serum-induced cellular stress, H_2_O_2_ and anticancer-drug-induced stresses. Most of these stresses contribute to generate reactive oxygen species (ROS), leading to telomere erosion and DNA damage [1, 2, 5, 6]. Therefore, cells in culture provide an excellent *in vitro* model for cellular senescence study and to delineate its molecular basis.

Although common senescence markers or regulators play a key role in stress-induced and replicative senescence, not all major senescence markers are expressed in every cell type undergoing senescence [1, 2, 5-7]. A recent study on gene expression profiling of replicative and different stress-induced senescence suggests that major markers and regulators of senescence are common in both replicative and stress-induced senescent cells [7]. The key features of senescent cells are: irreversible growth arrest, flattened morphology, senescence-associated-beta-galactosidase (SA-β-gal) expression, elevated expression of cell cycle inhibitors like p16, p21 and p53, growth factor TGF-β, growth factor binding protein IGFBP3 and serine protease inhibitor PAI-1 [8-11]. We and others have established the significant role of PAI-1 in stress and aging-associated cellular senescence as well as in development of numerous human diseases including cardiovascular and renal diseases [8-17]. The present study was undertaken to test the hypothesis that pharmacological inhibition of PAI-1 activity may protect normal cells from stress-induced and aging-associated cellular senescence. We were also interested in delineating the involvement of different senescence regulators in three major cell types and mode of action of PAI-1 inhibitor in these pathways.

In order to test our hypothesis we investigated the role of a small molecule inhibitor of PAI-1, TM5441 in different stress activated cellular senescence processes with special emphasis on Doxorubicin. Doxorubicin or Adriamycin belongs to anthracycline family of antibiotics. It has been an important component of various tumor therapies including leukemias, osteosarcomas and mesotheliomas as it negatively affects the activity of topoisomerases by intercalating between the base sequences of DNA [18, 19]. However, the major drawback of this drug is its cardiotoxic side effects that lead to cardiomyopathy characterized by abnormal heart function and development of cardiac fibrosis. Doxorubicin induces cellular senescence and eventually drives the cells to the death pathway [18-21]. It is known that PAI-1 is an important regulator of cellular senescence and importantly PAI-1 is induced by Doxorubicin *in vitro* in cultured cells and in cancer patients undergoing Doxorubicin treatment [11, 20]. These important findings lead us to test the hypothesis that pharmacological inhibition of cellular PAI-1 activity using a specific inhibitor protects cells from doxorubicin-induced senescence, and its associated complications.

In the present study, we tested the efficacy of a small molecule TM5441, a potent inhibitor of PAI-1, in prevention of stress and aging associated cellular senescence using different cell types. Our results suggest that TM5441 has protective effect on stress-induced and aging-induced cellular senescence via upregulation of ROS quenchers like antioxidant catalase and suppression of senescence regulators p16-p21-p53-PAI-1 and IGFBP3 signaling pathways. Therefore, PAI-1 is a druggable target and pharmacological inhibition of elevated PAI-1 levels may protect healthy cells from stress-induced premature senescence and accelerated aging process.

## RESULTS

### TM5441 inhibits Doxorubicin-induced cellular senescence characterized by morphology and SA-β-gal assay

**Figure 1:**
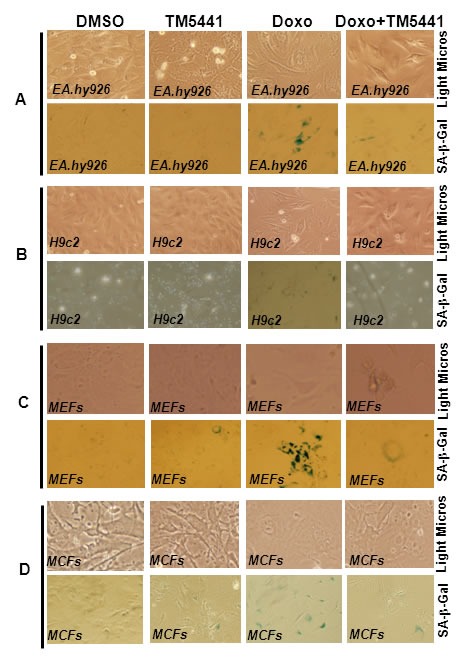
PAI-1 inhibitor blunts Doxorubicin-induced senescence in endothelial cells, cardiomyocytes, and fibroblasts Cultures of cells were pretreated with TM5441 for 24 h followed by Doxorubicin (Doxo) treatment for 4 days. At the end of incubation, morphological images were captured by light microscopy (**A.**-**D.**, upper panel). The cells were subjected to SA-β-gal assay and photographed (**A**-**D**, lower panel). Images are representative of three independent treatments. SA-β-gal images in **A**, **C** and **D** (lower panel) are representative part of Supplemental Figure 1A, C and D. For details see supplemental data Figure 1A-D.

We have tested the effect of a novel small molecule PAI-1 inhibitor, TM5441, on Doxorubicin-induced cellular senescence in three major cell types. Doxorubicin is a chemotherapeutic agent that induces cellular senescence via inhibition of Topoisomerase II and DNA damage [18-21]. Cultured cells (cardiomyocytes, fibroblasts and endothelial cells) were pretreated with TM5441 for 1 day followed by treatment with Doxorubicin for 4 days. Cellular senescence was confirmed by morphological changes and or SA-β-gal assay in these cell types. PAI-1 inhibitor TM5441-treated cells were morphologically comparable to vehicle (DMSO) treated cells. Doxorubicin treated endothelial cells and cardiomyocytes are morphologically more flattened, a characteristic of senescent cells. However, co-treatment with TM5441 reduces Doxorubicin-induced morphological changes (Figure 1A-1D, upper panels). Mouse embryonic fibroblasts (MEFs) and mouse cardiac fibroblasts (MCFs) are morphologically not distinct from control and treated groups. SA-β-gal assay of control and treated groups show that while Doxorubicin induces senescence in endothelial cells and fibroblasts as evidenced by the presence of significant number of SA-β-gal positive cells, very few H9c2 cells are SA-β-gal positive under the same experimental condition. Most importantly, TM5441 pretreated cells are significantly protected from Doxorubicin-induced cellular senescence as evidenced by the decrease in SA-β-gal positive cells in cultures co-treated with Doxorubicin and TM5441 (Figure 1A-1D lower panels; see also Supplemental Figure 1A-1D). The levels of SA-β-gal positive cells in endothelial, fibroblast and cardiomyocyte cultures treated with TM5441 alone are comparable with that of vehicle DMSO treated cells. To determine the effects of Doxorubicin and TM5441 on cellular proliferation, cardiac fibroblasts were pretreated with TM5441 followed by Doxorubicin treatment for 24 h. At the end of incubation, cellular proliferation was assessed. Data reveal that Doxorubicin significantly inhibits cellular proliferation and TM5441 partially blocks Doxorubicin-induced inhibition of cellular proliferation (Supplemental Figure 2A, 2B).

### TM5441 blocks Doxorubicin-induced cellular senescence characterized by senescence regulators

**Figure 2:**
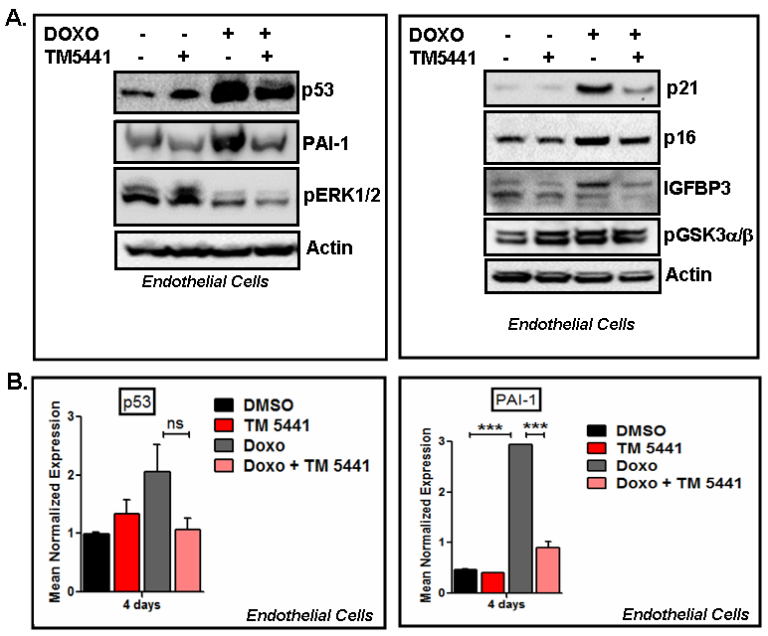
PAI-1 inhibitor TM5441 inhibits Doxorubicin-induced senescence regulators in endothelial cells Cultures of human endothelial cells EA.hy926 were pretreated with TM5441 (10 μM) for 24h followed by Doxorubicin (Doxo) treatment in triplicate for 4 days. Total proteins and RNA were collected from three independently treated wells and pooled. Experiments were repeated two times. Proteins were analyzed for senescence markers and regulators by Western blot using specific antibodies as indicated **A.** Total RNA extracted from control and treated groups were subjected to qRT-PCR analysis using gene specific primers. Data are presented as mean ± sem. *** denotes *p* = 0.0001. **B.**

In order to delineate the molecular basis of Doxorubicin-induced cellular senescence and the protective effect of TM5441, we next examined the levels of senescence regulators in protein lysates prepared from control, TM5441 alone, Doxorubicin alone and Doxorubicin plus TM5441 treated cells by Western blot. Data shows that Doxorubicin significantly induces the levels of potent senescence inducers p53, IGFBP3, PAI-1, p16 and p21 in human endothelial cells (Figure 2A). Most importantly, treatment with PAI-1 inhibitor TM5441 inhibits the expression levels of p53, PAI-1, p16, p21 and IGFBP3, the major regulators of cellular senescence in endothelial cells. Our results show that TM5441 mediated inhibition of Doxorubicin-induced p53 is modest. Next we tested the effect of TM5441 on Doxorubicin-induced senescence regulators p53 and PAI-1 at the transcript levels. Hence, consistent with the protein data we observed that Doxorubicin significantly induces PAI-1 mRNA in endothelial cells. While the Doxorubicin-mediated induction of p53 mRNA and its suppression by TM5441 are non-significant (*p* = 0.15), TM5441 significantly suppresses Doxorubicin-induced of PAI-1 mRNA expression (*p* = 0.0001) indicating suppression is regulated at the transcript level (Figure 2B). To determine the effect of TM5441 on early stage of Doxorubicin-induced endothelial senescence, we measured the protein levels of p53 and PAI-1. Data show that after 24h treatment of Doxorubicin, the levels of p53 and PAI-1 proteins are significantly elevated at early time point and TM5441 suppresses the basal as well as Doxorubicin-induced elevated levels of p53 and PAI-1 (Supplemental Figure 3). Therefore, our data clearly predicts that pretreatment with TM5441 reduces Doxorubicin-induced cellular senescence. Since high concentration of Doxorubicin induces significant DNA damage, we asked whether low concentration of Doxorubicin (50 nM) (in our experimental conditions) affects DNA integrity. We observed that while 50 nM Doxorubicin inhibited cellular proliferation and induced senescence markers like p53 and PAI-1, no appreciable DNA damage was observed within 24 h. TM5441 had no effect on the integrity of genomic DNA as well (Data not shown).

**Figure 3:**
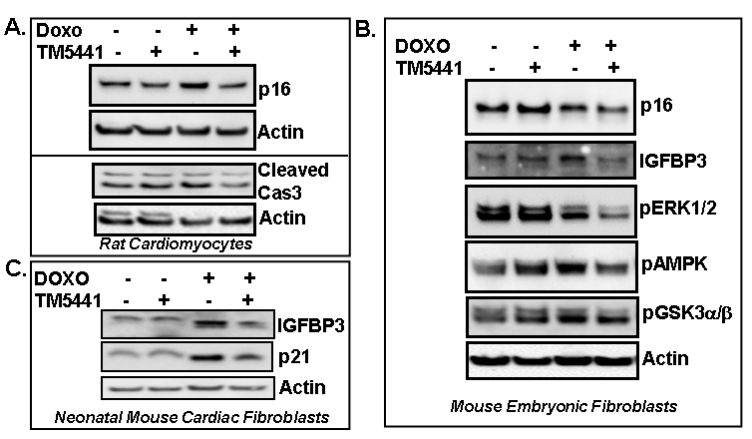
PAI-1 inhibitor TM5441 inhibits Doxorubicin-induced senescence regulators in cardiomyocytes and fibroblasts Cultures of rat cardiomyocytes H9c2 **A.**, mouse embryonic fibroblasts (MEFs) **B.** and neonatal mouse cardiac fibroblasts (MCFs) **C.** were pretreated with TM5441 for 24h followed by Doxorubicin (Doxo) treatment in triplicate for 4 days. Total proteins were collected from three independently treated wells and pooled. Experiments were repeated two times. Proteins were analyzed for senescence markers and regulators by Western blot using specific antibodies as indicated.

As ERK1/2 MAPK and GSK3α/β are known to be involved in cellular senescence in certain cell types [5, 6], we examined the level of pERK1/2 MAPK in Doxorubicin-induced senescent cells. Results show that TM5441 fails to block Doxorubicin-induced suppression of ERK1/2 MAPK activation in endothelial cells (Figure 2) indicating ERK1/2MAPK is not involved in TM5441-mediated inhibition of Doxorubicin-induced cellular senescence. The levels of pGSK3α/β are not altered by the presence of TM5441 in endothelial cells (Figure 2). Next the efficacy of TM5441 in prevention of Doxorubicin-induced senescence in rat cardiomyocytes was investigated. In H9c2 cells, the level of senescence regulator p16 protein is elevated by Doxorubicin compared to basal level. Importantly, PAI-1 inhibitor TM5441 significantly reduces the Doxorubicin-induced elevated level of p16 in rat cardiomyocytes H9c2 (Figure 3A upper panel). Doxorubicin treatment causes modest increase in cleaved caspase3 (active form) levels in H9c2. However, pretreatment with TM5441 reduces the levels of cleaved caspase3 in Doxorubicin treated H9c2 (Figure 3A lower panel). We further investigated the effect of PAI-1 inhibitor TM5441 on Doxorubicin-induced senescence in fibroblasts and the underlying molecular basis. Western blot analysis of protein extracts for senescence regulators show that while IGFBP3 level is stimulated by Doxorubicin in mouse embryonic fibroblasts (MEFs), the level of p16 remains unaltered compared to basal level of p16. However, PAI-1 inhibitor TM5441 decreases the potent senescence regulators p16 and IGFBP3 in Doxorubicin treated MEFs (Figure 3B). In primary cultures of neonatal mouse cardiac fibroblasts, Doxorubicin induces the levels of p21 and IGFBP3 and the Doxorubicin-induced stimulation of p21 and IGFBP3 levels are significantly decreased by TM5441 indicating PAI-1 inhibition efficiently blocks fibroblast senescence (Figure 3C). In order to understand the signaling pathways involved in TM5441-mediated suppression of Doxorubicin-induced fibroblast senescence, we examined ERK1/2-MAPK, AMPK and GSK3α/β pathways which are known to be involved in Doxorubicin-induced senescence and apoptosis [1, 2, 5, 6]. Doxorubicin inhibits pERK1/2 MAPK levels. However, TM5441 fails to block Doxorubicin-induced inhibition of ERK1/2 MAP kinase activation in MEFs (Figure 3B). The levels of pAMPK and pGSK3α/β are modestly inhibited by TM5441 in the presence of Doxorubicin in fibroblasts (Figure 3B).

### TM5441 blunts Doxorubicin-mediated suppression of catalase in endothelial cells

**Figure 4:**
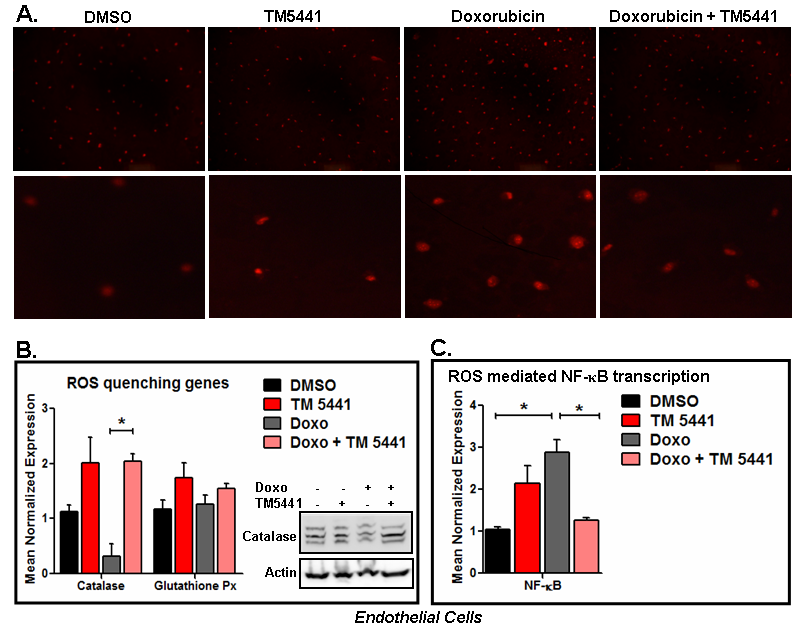
PAI-1 inhibitor TM5441 inhibits Doxorubicin-induced ROS generation in endothelial cells EA.hy926 endothelial cells were pretreated with TM5441 followed by Doxorubicin treatment in triplicate for 2 h. Experiments were repeated two times. The levels of oxidized superoxide were determined using MitoSox kit and photographed (upper panel 10X and lower panel 20X) **A.** Batches of endothelial cells were pretreated with TM5441 followed by Doxorubicin (Doxo) treatment for 4 days in triplicate. Total RNA isolated from control and treated cells. The levels of ROS quenching factors and ROS target genes were measured by qPCR using gene specific primers. * denotes *p* = 0.028 (**B**) and *p* = 0.024 (**C**) **B.**, **C.** Whole cell lysates were prepared from three wells for each group and pooled. Experiments were repeated two times. The levels of catalase protein in control and treated endothelial cells were measured by Western blot (**B**).

As endothelial cells are the major source of PAI-1 and involved in different cardiovascular diseases [8-17], we choose to use endothelial cells to study the effect of PAI-1 inhibitor on Doxorubicin-induced ROS and ROS quenching factors. Reactive oxygen species (ROS) play a major role in stress-induced diseases and aging. ROS are produced in both mitochondria and non-mitochondrial sources as by-products of different metabolic processes in stressed cells. Several endogenous enzymes including catalase, glutathione peroxidase, superoxide dismutase (SOD) are involved in protection of ROS-induced cellular damage [22]. Here we first determine the effect of TM5441 on Doxorubicin-induced ROS generation using MitoSOX™ Red mitochondrial superoxide indicator for live-cell imaging. Data in endothelial cells show that Doxorubicin induces the level of ROS production and TM5441 significantly reduces the level of ROS as evidenced by the level of red fluorescence images of superoxide-mediated oxidized MitoSOX™ Red reagent (*p* < 0.05) (Figure 4A and Supplemental Figure 4A, 4B).

Next we determined the effect of TM5441 on mRNA expression levels of ROS quenching molecules catalase, SOD and glutathione peroxidase in Doxorubicin treated endothelial cells. The qPCR analysis of total RNA isolated from control and treatment groups demonstrate that while Doxorubicin suppresses the expression of ROS quenching factor catalase, TM5441 induces the basal level of catalase and blunts Doxorubicin-mediated suppression of catalase mRNA expression (*p* = 0.028) (Figure 4B). Like catalase mRNA, Doxorubicin also reduces the levels of catalase protein in endothelial cells and fails to inhibit catalase protein in the presence of PAI-1 inhibitor TM5441 (Figure 4B). Under the same experimental condition, the level of Glutathinone peroxidase mRNA expression is not significantly affected by Doxorubicin and TM5441 in this endothelial cell line (Figure 4B). We also noticed that SOD mRNA level is upregulated by Doxorubicin and TM5441 suppresses the increased expression of SOD by Doxorubicin. TM5441 alone modestly induces SOD basal expression (Supplemental Figure 4C). We suspect that the elevated level of SOD in the presence of Doxorubicin may be due to an adaptive response. As TGF-β is also a SASP or SMS [1, 2], we have determined the level of TGF-β1 mRNA. The level of TGF-β1 mRNA is not affected by the presence of Doxorubicin. However, TM5441 reduces the level of TGF-β1 mRNA in the presence of Doxorubicin (Supplemental Figure 4D). Next we have determined the effect of Doxorubicin and TM5441 on expression level of NFκB. NFκB is one of the critical nuclear factors upregulated by enhanced oxidative stress and plays a key role in senescence [23]. Our data show that Doxorubicin induces the level of NFκB and TM5441 suppresses that induction (Figure 4C).

### TM5441 blocks stress-induced and replicative senescence in fibroblasts

**Figure 5:**
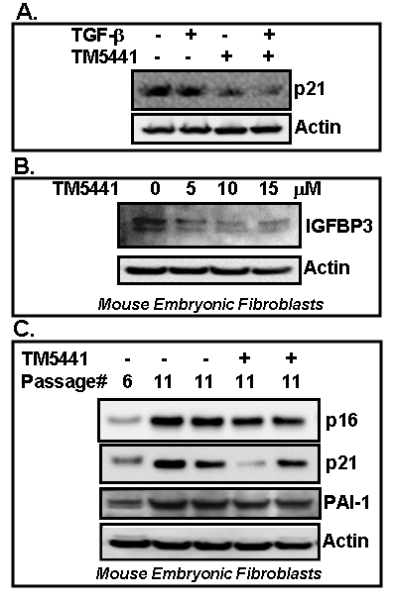
PAI-1 inhibitor TM5441 inhibits stress-induced and replicative senescence in fibroblasts MEFs were cultured in the presence or absence of TGF-β (**A**) or for longer period of time (7 days) (**B**, culture shock). Batches of cells were treated with PAI-1 inhibitor TM5441 in triplicate. Cell lysates were processed for Western blot using specific antibodies as indicated **A.**, **B.** For replicative senescence study, cultures of mouse embryonic fibroblasts were treated with vehicle or TM5441 (10 μM) for several passages in duplicate. Cell extracts were prepared from passage 6 (early) and 11 (late, with or without TM5441). Proteins were analyzed for senescence regulators p16, p21 and PAI-1 by Western blot **C.**

In order to test the efficacy of PAI-1 inhibitor TM5441 on different cellular stress-induced senescence markers, we have also tested the effect of TM5441 in cellular senescence in response to TGF-β and *in vitro* culture shock in mouse embryonic fibroblasts. Mouse embryonic fibroblasts were treated with TGF-β or cultured for prolonged period in the presence or absence of small molecule TM5441. Cell lysates were prepared and subjected to Western blot for senescence markers. Protein analysis results indicate that under this experimental condition, the level of p21 is not induced by TGF-β, however PAI-1 inhibitor TM5441 efficiently reduces the levels of potent senescence regulators p21 and IGFBP3 in TGF-β treated and culture-shocked MEFs respectively (Figure 5A, 5B). Next we examined the effect of PAI-1 inhibitor TM5441 on cellular replicative senescence in mouse embryonic fibroblasts. MEFs (passage 6) were treated with or without TM5441 and passaged up to 11. Whole cell extracts were prepared from passage 6 (as early passage) and passage 11 (as late passage) in the presence and absence of TM5441. Western blot analysis results show that the levels of senescence markers p21 and p16 are significantly elevated in passage 11 (late) MEFs compared to passage 6 (early) MEFs. However, the levels of p16 and p21 were reduced in passage 11 cells grown in the presence of TM5441 (10 μM from passage 6 to 11) (Figure 5C). The levels of PAI-1 are elevated in higher passage compared to early passage. Presence of TM5441 partially blocks the level of PAI-1 protein in fibroblasts. These results indicate that PAI-1 inhibitor TM5441 is effective in blocking aging-associated or replicative cellular senescence.

### TM5484, another potent inhibitor of PAI-1, inhibits cellular senescence

**Figure 6:**
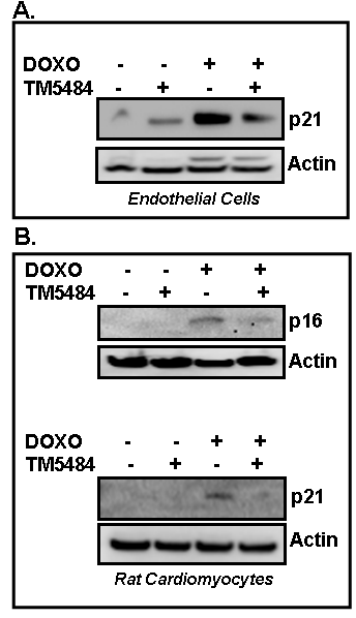
PAI-1 inhibitor TM5484 inhibits Doxorubicin-induced senescence in cardiomyocytes and endothelial cells Cultures of endothelial cells **A.** and cardiomyocytes H9c2 **B.** were pretreated in triplicate with TM5484 for 24h followed by Doxorubicin (Doxo) treatment in triplicate for 4-5 days. Proteins were analyzed for senescence markers and regulators as indicated by Western blot using specific antibodies.

Next we tested the efficacy of another small molecule TM5484, a potent inhibitor of PAI-1, in suppression of Doxorubicin-induced cellular senescence in endothelial cells (Figure 6A) and cardiomyocytes (Figure 6B). Cells were pretreated with TM5484 for 24 h followed by Doxorubicin treatment for 4 days. At the end of treatment, cells were harvested and cell lysates were prepared and subjected to Western blot. Results reveal that like TM5441, TM5484 also significantly inhibits Doxorubicin-induced senescence regulator p16 and p21 in endothelial cells and cardiomyocytes (Figure 6A, 6B).

## DISCUSSION

Plasminogen Activator Inhibitor-1 (PAI-1) plays a pivotal role in cellular senescence during aging and under pathological milieu. While physiological level of PAI-1 is essential for maintenance of normal cellular and physiological homeostasis, complete loss or super-physiological levels of PAI-1 are detrimental for cellular and physiological processes. Elevated levels of PAI-1 are associated with aging and numerous human diseases including cardiovascular diseases [12-17]. Recently, we have demonstrated that genetic or pharmacological inhibition of PAI-1 activity increases the lifespan in a murine model of accelerated aging [15]. Importantly, genetically ablated PAI-1 activity in this accelerated aging murine model (*klotho* deficient mice) is associated with decreased levels of a senescence regulator p16^Ink4a^ and increased life span [15] indicating enhanced cellular senescence is a significant contributor in accelerated aging process. In order to prevent premature cellular senescence associated with accelerated aging process, we examined the effect of a druggable small molecule-mediated inhibition of stress or aging-associated elevated levels of PAI-1 in prevention of cellular senescence in three major cell types, cardiomyocytes, endothelial cells and fibroblasts.

The major findings of the present study provide substantial evidences in favor of the protective role of PAI-1 inhibitor TM5441 on Doxorubicin-induced and replicative senescence and underpinning molecular basis. We choose to study the effect of TM5441 on Doxorubicin-induced cellular senescence using three major cell types. The rationale behind using Doxorubicin as a senescence stressor in the present study was twofold. First, Doxorubicin generates reactive oxygen species (ROS) the major mediator of cellular senescence; Second, while Doxorubicin is an effective anticancer drug, its major downside is its cardiotoxic side-effects due to DNA intercalation leading to cellular senescence, apoptosis, cardiomyopathy and ultimate death in a high percentage of patients treated with this drug [19]. Therefore, identification of a druggable small molecule inhibitor of senescence is an important task to prevent senescence and apoptosis-associated cardiomyopathy and abnormal cardiac function. As Doxorubicin induces the levels of PAI-1, as evidenced by *in vitro* studies as well as in patients treated with anticancer drug [11, 20], inhibition of elevated PAI-1 levels using specific inhibitor may protect patients from Doxorubicin-induced cellular abnormality in healthy cells. The present study identified that PAI-1 inhibitor TM5441 blocks both stress-activated and replicative cellular senescence via suppression of a major pool of senescence regulators including p16, p21, p53, IGFBP3 and PAI-1. It is important to note that the extent of TM5441 mediated suppression of senescence regulators are cell-type specific. The next obvious question is how PAI-1 inhibitor TM5441 blocks the induction of different senescence mediators or regulators. We propose that beneficial effect of TM5441 stems from its ability to prevent Doxorubicin-induced catalase suppression. Increased catalase expression reduces oxidative stress on endothelial cells and reduces stress induced premature senescence (SIPS). This notion is in agreement with previous observation that overexpressed catalase inhibits redox-induced PAI-1 expression [24], and also protects hearts from Doxorubicin-induced cardiotoxicity [21, 25, 26]. Secondly, the cell cycle regulator and senescence mediator p53 is also known to be induced by DNA damage and is a known inducer of both PAI-1 and cell cycle inhibitor p21. Although p53 is a bonafide inducer of cellular senescence, Leontieva et al. [27] demonstrated that while low levels of p53 induces geroconversion, high levels of p53 causes gerosuppression via inhibition of mTOR. As overexpressed PAI-1 mediates cellular senescence even in the absence of p53 [9], it is reasonable to speculate that cellular PAI-1 exerts its pro-senescence activity directly or indirectly via modulation of other important senescence regulators.

**Figure 7:**
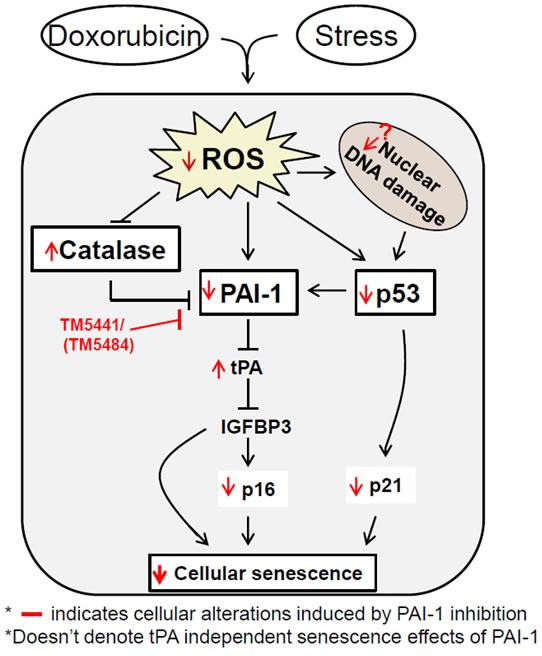
Model depicting the possible molecular basis of TM5441-mediated suppression of cellular senescence Cellular stressors induce ROS that is associated with induction of kinases and cell cycle regulators which dictate the cells to undergo senescence. The individual senescent regulator functionally interacts with different other regulators like p53, p16, p21, IGFBP3, PAI-1 and participate in cellular senescence process in a cell type or stressor-dependent manner. Inhibition of cellular PAI-1 activity using specific small molecule inhibitor and activation of tPA/uPA axis are able to disrupt this senescent regulatory network and senescence in all three major normal cell types.

PAI-1 inhibitor TM5441-mediated suppression of ROS may at least partially inhibit p53 expression and that may affect expression of p53 target genes p21 and PAI-1. Similarly, inhibition of PAI-1 protein level in TM5441 treated cells may be due to suppression of ROS generation because ROS augments PAI-1 expression [28]. Thirdly, another important senescent regulator IGFBP3 is a downstream target of PAI-1-tPA axis where tPA inhibits IGFBP3 by proteolysis [11]. Using an unbiased analysis of SASP, recently Ozcan et al. [29] also identified IGFBP3-PAI-1 is one of the key signaling pathways involved in cellular senescence in response to different genotoxic stressors. The inhibition of Doxorubicin-induced senescence regulator IGFBP3 by TM5441 may be due to activated tPA-mediated proteolysis of IGFBP3 in the absence of active PAI-1. As IGFBP3 is a positive regulator of cell cycle regulator p16, TM5441-mediated suppression of p16 is likely due to decreased level of IGFBP3 (Figure 7). It is important to note that a recent study demonstrated that TM5441 decreased the viability of different cancer cell lines and reduced tumor growth. However, TM5441 did not potentiate the cytotoxicity of Doxorubicin in these cancer cell lines [17]. Therefore, these results together suggest the usefulness and significance of PAI-1 specific safe druggable small molecule inhibitor in preventing senescence of healthy cells during exposure to chemotherapeutic drug Doxorubicin and also during aging process.

Lastly, we also investigated whether TM5441 mediated suppression of Doxorubicin-induced senescence is due to alteration of specific kinase pathway that is known to be involved in cellular senescence. Previous studies established the involvement of MAPKs in senescence and apoptosis in different cell types. For example, ERK1/2 MAPK, p38 MAPK, AMPK and AKT play a role in the regulation of cellular senescence induced by a wide variety of stressors including hydrogen peroxide, *in vitro* culture shock, Doxorubicin treatment etc. [1, 2, 5, 6, 30]. Additionally, activation or inhibition of these kinases blocks doxorubicin-induced cardiomyocyte and other cellular dysfunction [31-36]. Here, we examined the activity of ERK1/2 MAPK, AMPK and GSK-3α/β signal transducers in Doxorubicin and PAI-1 inhibitor TM5441 treated cells. Present study showed that while the levels of pERK1/2 MAPK is dramatically abrogated by Doxorubicin, pAMPK levels were modestly affected. The Doxorubicin-induced inhibition of pERK1/2 MAPK was not rescued by TM5441 treatment indicating ERK1/2 MAPK may not be involved in the TM5441-mediated inhibition of Doxorubicin-induced cellular senescence. The present study also demonstrated that TM5441 modestly inhibits the levels of pGSK3α/β in endothelial cells treated with Doxorubicin indicating that TM5441 may exerts its rescue effect on Doxorubicin-induced endothelial cellular senescence via suppression of GSK3α/β activation.

In conclusion, we have discovered that PAI-1 inhibitor TM5441 efficiently decreases the levels of senescence in three major normal cell types i.e., cardiomyocytes, fibroblasts and endothelial cells via suppression of major senescence regulators p16, p21, p53, IGFBP3 and PAI-1 in a cell-type specific manner (Figure 7). These cadres of cellular senescence are known to induce senescence individually as well as coordinately based on cell type and nature of stressors or insults and aging process as discussed earlier. Based on our results in vascular endothelial cells, it is reasonable to conclude that suppression of ROS generation and activation of antioxidant catalase play a pivotal role in TM5441-mediated suppression of cellular senescence. The beneficial inhibitory effect of TM5441 on senescence in these normal cells may be due to suppression of specific signaling pathway and specific senescence regulator in a cell type dependent manner. Therefore, it is reasonable to infer that suppression of PAI-1 using small molecule inhibitor is an ideal approach to prevent Doxorubicin-induced premature senescence of different cells and aging associated cellular abnormality. Future study using animal model is necessary to validate the protective role of PAI-1 inhibitor on Doxorubicin-induced cellular senescence, cardiomyopathy and replacement cardiac fibrosis.

## MATERIALS AND METHODS

### Cell culture and treatment with senescence inducers and PAI-1 inhibitors

Rat cardiomyoblast cells H9c2 proliferate in culture and serves as an animal-free alternative. H9c2 cells and primary rat neonatal cardiomyocytes show almost identical hypertrophic responses when exposed to Angiotensin II and Endothelin-1 based on average cellular area/hypertrophy and expression of F-actin, s-α-actinin and BNP [37]. Human endothelial cells EA.hy926 is derived by fusing HUVEC and human lung carcinoma cell line A549. This cell line possesses endothelial characteristics. [38]. Mouse embryonic fibroblasts (MEFs), rat neonatal cardiomyocytes H9c2, human cardiac fibroblasts and human endothelial cells EA.hy926 were purchased from ATCC (Manassas, VA). Neonatal mouse cardiac fibroblasts were isolated from neonatal wildtype mice and primary cultures were established. All cells were grown in DMEM containing 10% fetal bovine serum and 1% penicillin and streptomycin and maintained at 37°C in a 5% CO_2_ incubator. Confluent cultures were typsinized and 50000-100000 cells/well were subcultured in 12 well-clusters and were pretreated with TM5441 or TM5484 in triplicate for 24 h followed by Doxorubicin treatment for 1-4 days.

### SA-β-gal assay

The widely used biomarker for senescent cells is the SA-β-galactosidase activity. SA-β-gal activity is detectable at pH 6.0 in senescent cells (BioVision, Milpitas, CA). Beta-galactosidase activity is expressed from GLB1, the gene encoding lysosomal-galactosidase. Cardiomyocytes H9c2, endothelial cells EA.hy926, mouse embryonic fibroblasts (MEFs) and neonatal mouse cardiac fibroblasts (nMCFs) were cultured in 12-well culture plates and pretreated with TM5441 for 24 h followed by Doxorubicin (50 nM) treatment in triplicate for 4 days. At the end of treatment, cells were processed for SA-β-gal using Senescence Assay Kit (Catalog Cat#K320-250; BioVision, Milpitas, CA). In brief, culture medium was removed and cells were washed once with 1X PBS and fixed with fixative solution for 15 min at room temperature. After fixation, cells were washed twice with 1XPBS and staining solution containing staining supplement and X-gal was added to each well and mixed. Plates were covered with aluminum foil and incubated overnight at 37°C without CO_2_. The SA-β-gal staining in cells were observed under a microscope for development of blue color and photographed.

### Induction of cellular senescence, protein extraction and western blot

Cardiomyocytes H9c2, endothelial cells EA.hy926, mouse embryonic fibroblasts (MEFs) and neonatal mouse cardiac fibroblasts (nMCFs) were cultured in 12-well culture plates and pretreated with TM5441 or TM5484 in triplicate (10 μM) for 24 h followed by Doxorubicin (50 nM) treatment for 1-4 days. At the end of indicated period of incubation, cells from three wells were harvested and pooled. For TGF-β treatment, MEFs were pretreated with TM5441 for 1h followed by TGF-β for 24 h. For culture shock, cells were grown for seven days and then treated with TM5441. Whole cell protein lysates were prepared using RIPA lysis buffer with protease and phosphatase inhibitors (Sigma, St. Louis, MO). Proteins were pooled from three control and treated wells. Equal amount of proteins were loaded on 4-12% Tris-glycine gradient gel and run at 90 volts for 2 h at room-temperature. The separated proteins were transferred to PVDF membrane using iBlot transfer apparatus (Invitrogen, Grand Island, NY). The PVDF membranes were blocked with 10% fat free milk containing TBST for 2 h at room temperature. Then membranes were incubated with antibodies against p16, p21, p53, IGFBP3, PAI-1, pERK1/2, pAMPK, pGSK3α/β and Actin overnight at 4°C. The membranes were washed followed by incubation with HRP-tagged specific secondary antibodies. The membranes were washed with TBST and the signals were detected by ECL reagents (Luminata Forte, Millipore, Billerica MA) and images were captured at BIORAD molecular imager ChemiDoc XRS system (BIORAD, Hercules, CA).

### Cell proliferation assay

Cardiac fibroblasts in 24-well culture plates were pretreated with TM5441 for 24 h followed by Doxorubicin treatment for 24 h (*n* = 6 wells). At the end of incubation, fibroblast proliferation was determined using CyQUANT-Direct assay kit according to manufacturer's instruction (Invitrogen, Grand Island, NY). Number of cells in control and treatment groups (*n* = 6 wells) were determined by ImagePro 9.1 software and presented as mean ± sem.

### DNA damage assay

The effect of TM5441 on Doxorubicin-induced DNA damage was determined by DNA fragmentation assay following published method [39]. The genomic was isolated from DMSO (control), TM5441 alone, Doxorubicin, and Doxorubicin+TM5541 combo treated endothelial cells using genomic DNA isolation kit (Qiagen). The extracted DNA from control and treated samples were subjected to electrophoresis on ethidium bromide dye containing 2 % agarose gel and was photographed in BIORAD imager under UV light.

### Quantitative qRT-PCR analysis

Total RNAs from control and treated cultured endothelial cells in triplicate were isolated and specific transcript level was determined by qPCR using gene specific primers. The messenger RNA (mRNA) levels of p53, PAI-1, catalase, glutathione peroxidase 1, NF-κB, SOD1, TGF-β and GAPDH were quantified by complementary DNA synthesis using iScript cDNA Synthesis Kit (Bio-Rad, Hercules, CA) and quantitative polymerase chain reaction with SYBR Green SuperMix for IQ (Quanta Bioscience, Gaithersburg, MD). Q-gene software was used for quantification. The qPCR primer sequences are as follows: *Catalase* For.: 5′-TCCGGGATCTTTTT AACGCCATTG-3′; Rev.: 5′-TCGAGCACGGTAGGGACAGTTCAC-3′. *PAI-1* For.: 5′-AAGACTC CCTTCCCCGACTC-3′; Rev.: 5′-GGGCGTGGTGAACTCAGTATAG-3′. *p53* For.: 5′GTTCCGAG AGCTGAATGAGG-3′; Rev.: 5′-TTATGGCGGGAGGTAGACTG-3′. *NF*-κ*B1* For.: 5′-AACAGAGAG GATTTCGTTTCCG-3′; Rev.: 5′-TTTGACCTGAGGGTAAGACTTCT-3′. *SOD1* For.: 5′-GGTCCT CACTTTAATCCTCTATCCAG-3′; Rev. 5′-CCAACATGCCTCTCTTCATCC-3′.*Glutathione peroxi dase 1* For.: 5′-GCGGCGGCCCAGTCGGTGTA-3′; Rev.: 5′-GAGCTTGGGGTCGGTCATAA-3′. *TGF-β1* For.: 5′-GGACA CCAACTATTGCTTCAG-3′; Rev.: 5′-TCCAGGCTCCAAATGTAGG-3′. *GAPDH* For.:5′-GAAGGTGAAGGTCGGAGT-3′; Rev: 5′-GAAGATGGTGATGGGATTTC-3′.

### ROS measurement

Human endothelial cells were pretreated with TM5441 (10 μM) for 24h followed by treatment with Doxorubicin (50 nM) in triplicate for 2 h. Cells were washed twice with HBSS and MitoSox Red fluorescent dye was used to assess mitochondrial O_2_production following manufacturer's protocol (Invitrogen, Carlsbad, CA). Cells were incubated with MitoSox Red at 37°C for 30 minutes. At the end of incubation cells were washed twice with HBSS. Stained cells in three independently treated wells were photographs in fluorescence microscope and analyzed. Red fluorescent positive cells were quantified using Image Pro-9.1 premier software (Media Cybernetics Inc., Rockville, MD).

### Statistical analysis

Data are presented as Mean ± SEM. The significance of differences between controls and experimental groups was estimated by one way ANOVA and the Turkey Multiple-comparison post hoc test. A value of*P* < 0.05 was considered statistically significant. Statistical analyses were performed with GraphPad Prism 3.0 (GraphPad Software Inc, San Diego).

## SUPPLEMENTARY MATERIALS FIGURES


