# Supplementary material for: A small molecule inhibitor of PAI-1 protects against doxorubicin-induced cellular senescence
Source: Oncotarget. 2016 Oct 6;7(45):72443–57. doi: 10.18632/oncotarget.12494 (PMC5341920; doi:10.18632/oncotarget.12494)
Supplement: Supplementary file 1 [file oncotarget-07-72443-s001.pdf]

A small molecule inhibitor of PAI-1 protects against Doxorubicin-induced cellular senescence: molecular basis

Supplementary Material

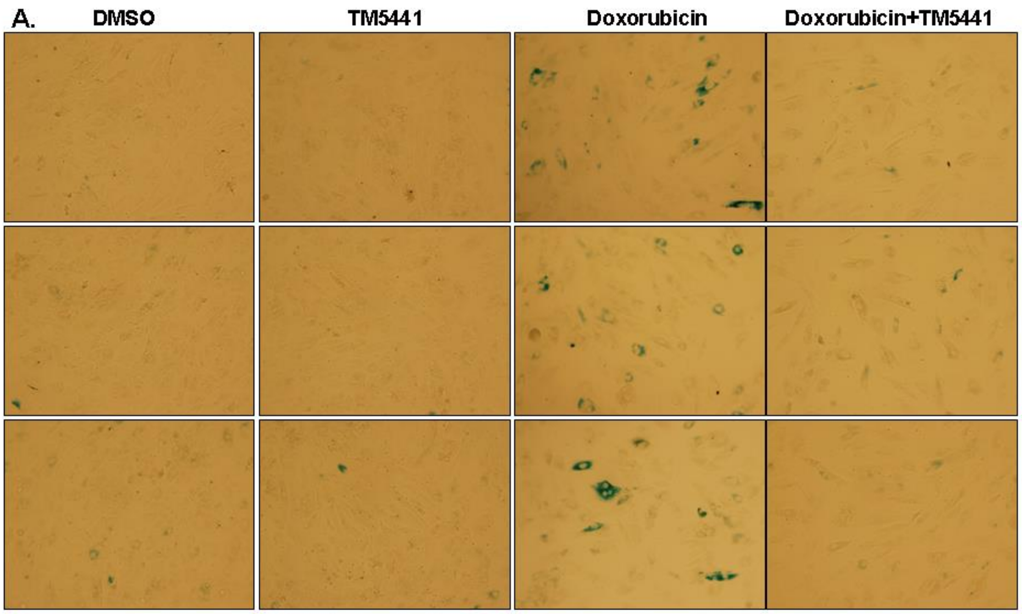

Endothelial Cells

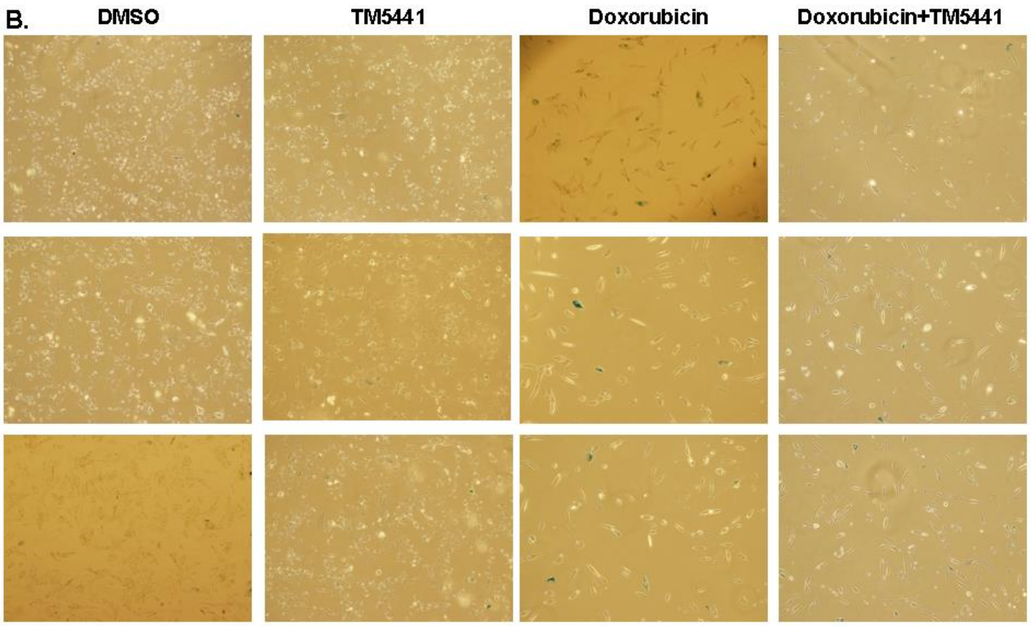

Rat Cardiomyocytes H9c2

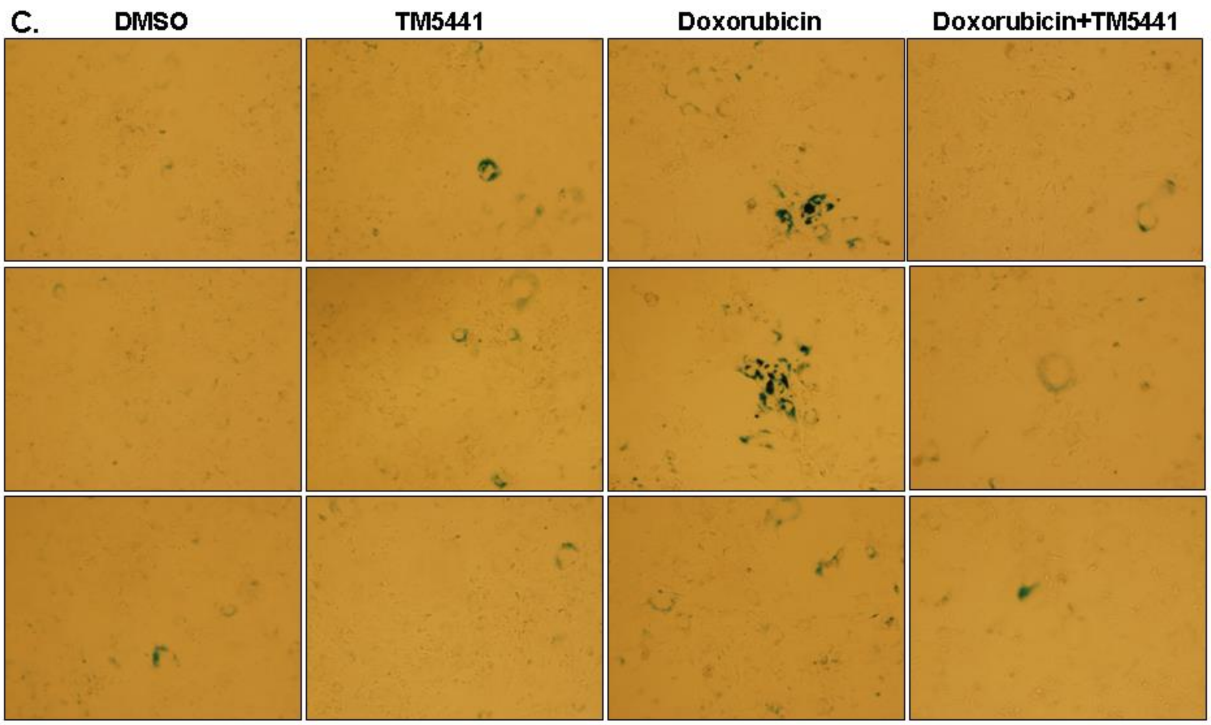

Mouse Embryonic Fibroblasts

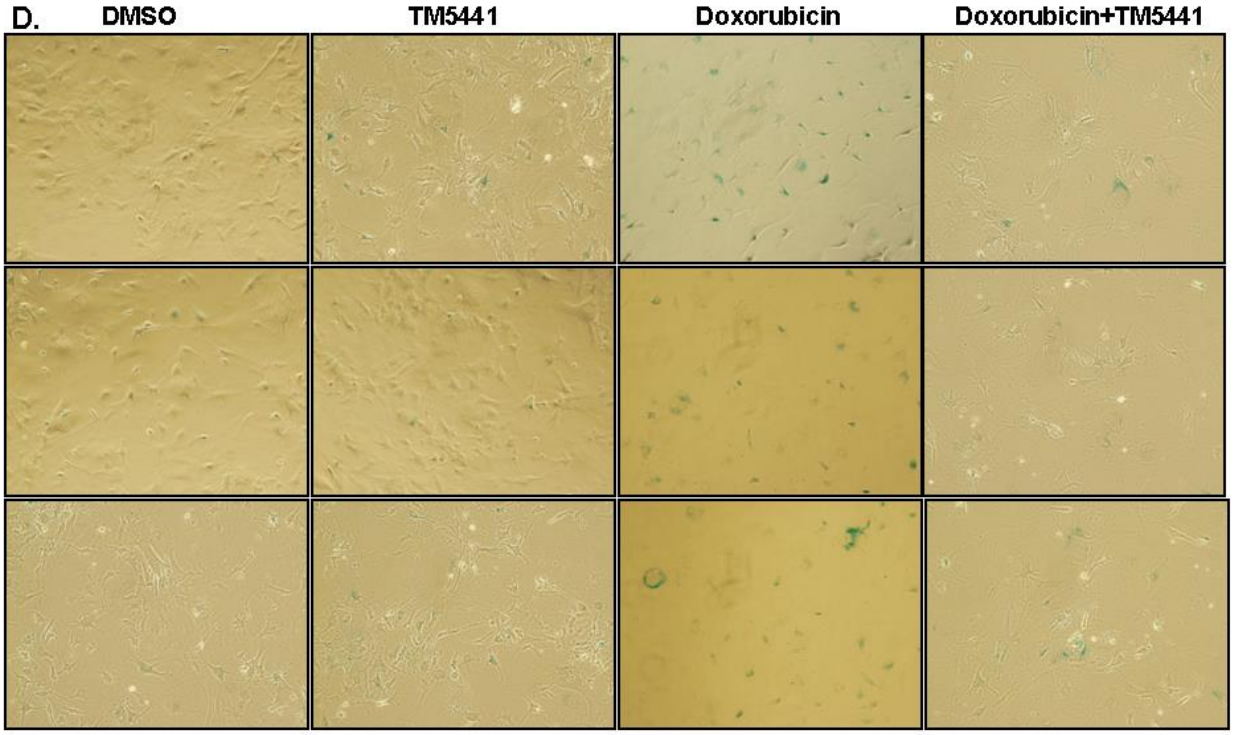

Neonatal Mouse Cardiac Fibroblasts

Supplemental Figure 1. PAI-1 inhibitor blunts Doxorubicin-induced senescence in endothelial cells, cardiomyocytes and fibroblasts: Cultures of human endothelial cells (**A**), rat cardiomyocytes (**B**), mouse embryonic fibroblasts (**C**) and primary cultures of mouse neonatal cardiac fibroblasts (**D**) were pretreated with TM5441 in triplicate for 24 h followed by Doxorubicin (Doxo) treatment for 4 days. At the end of incubation, cells were subjected to SA-β-gal assay and photographed (**A-D**). Part of A, C and D are presented in Figure 1A, C and D lower part.

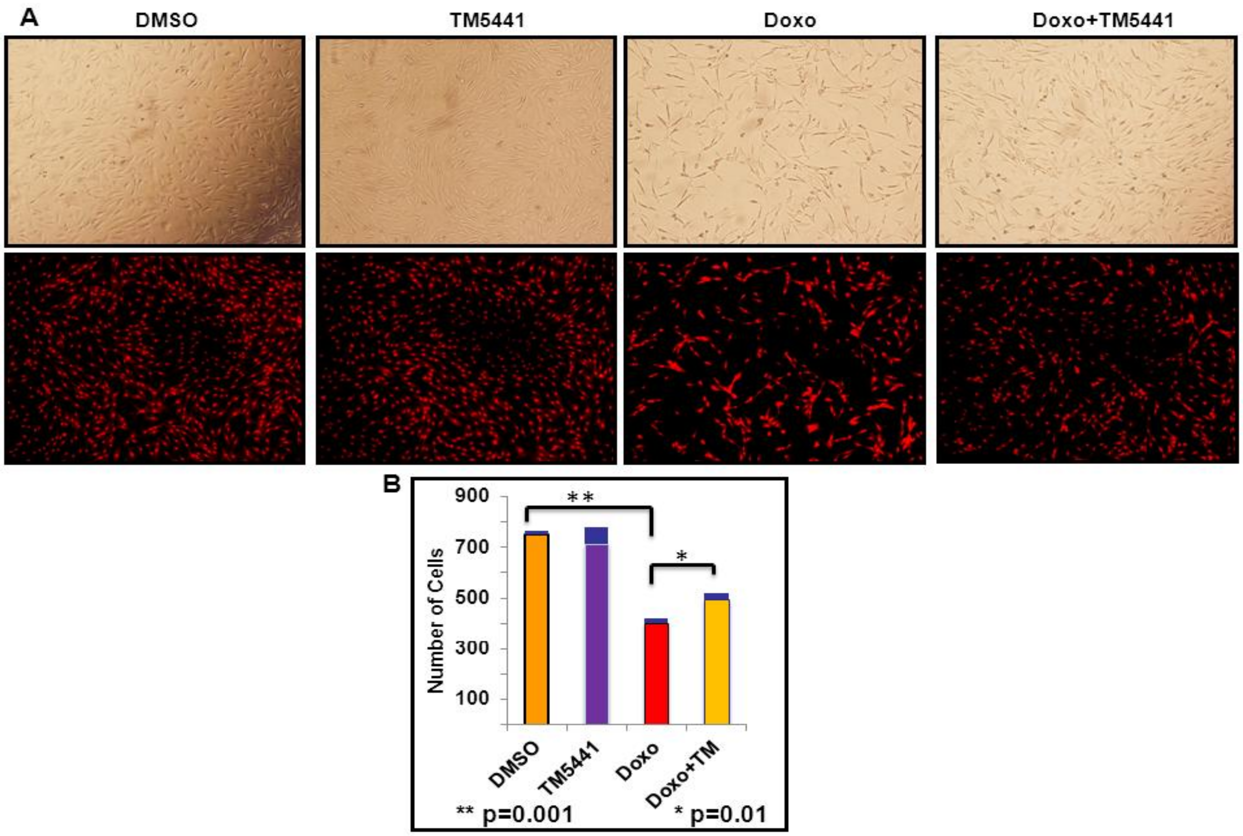

Supplemental Figure 2. Effect of TM5441 on Doxorubicin –induced inhibition of cellular proliferation. Cardiac fibroblasts were pretreated with TM5441 for 24 h followed by Doxorubicin treatment for 24h (n=6 wells). At the end of incubation, cells were subjected to proliferation assay using Direct CyQUANT kit (Invitrogen). Number of cells in control (n=6 wells) and treatment groups (n=6 wells) were presented as Mean ± SEM. ** p=0.001; * p=0.01.

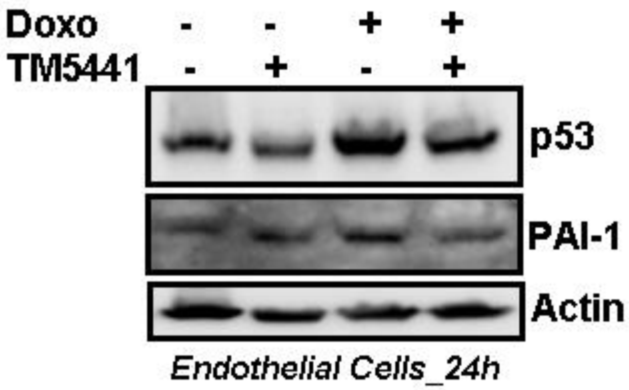

Supplemental Figure 3. Effect of TM5441 on Doxorubicin-induced senescence regulator at early time point. Cultures of human endothelial cells EA.hy926 were pretreated with TM5441 in triplicate for 24h followed by Doxorubicin (Doxo) treatment for 24h. Total protein isolated from triplicate control and treated groups were pooled and subjected to Western blot using p53, PAI-1 and Actin antibodies. Representative images of two experiments are shown.

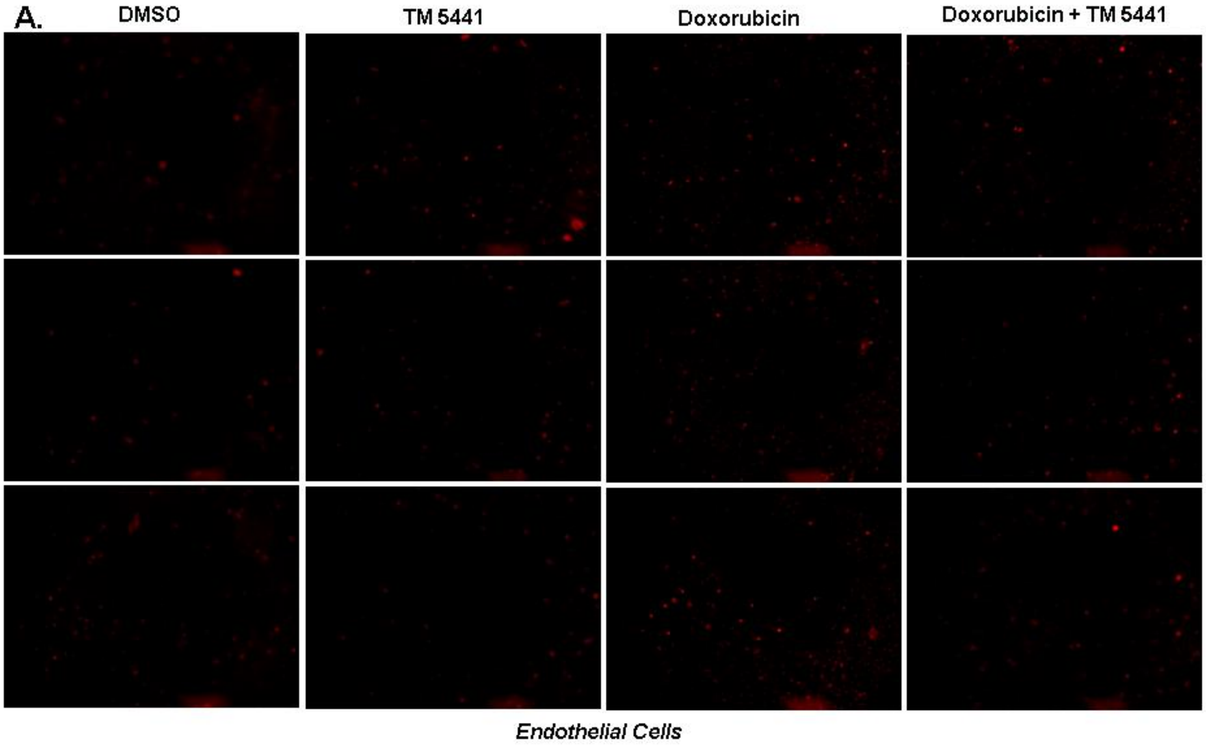

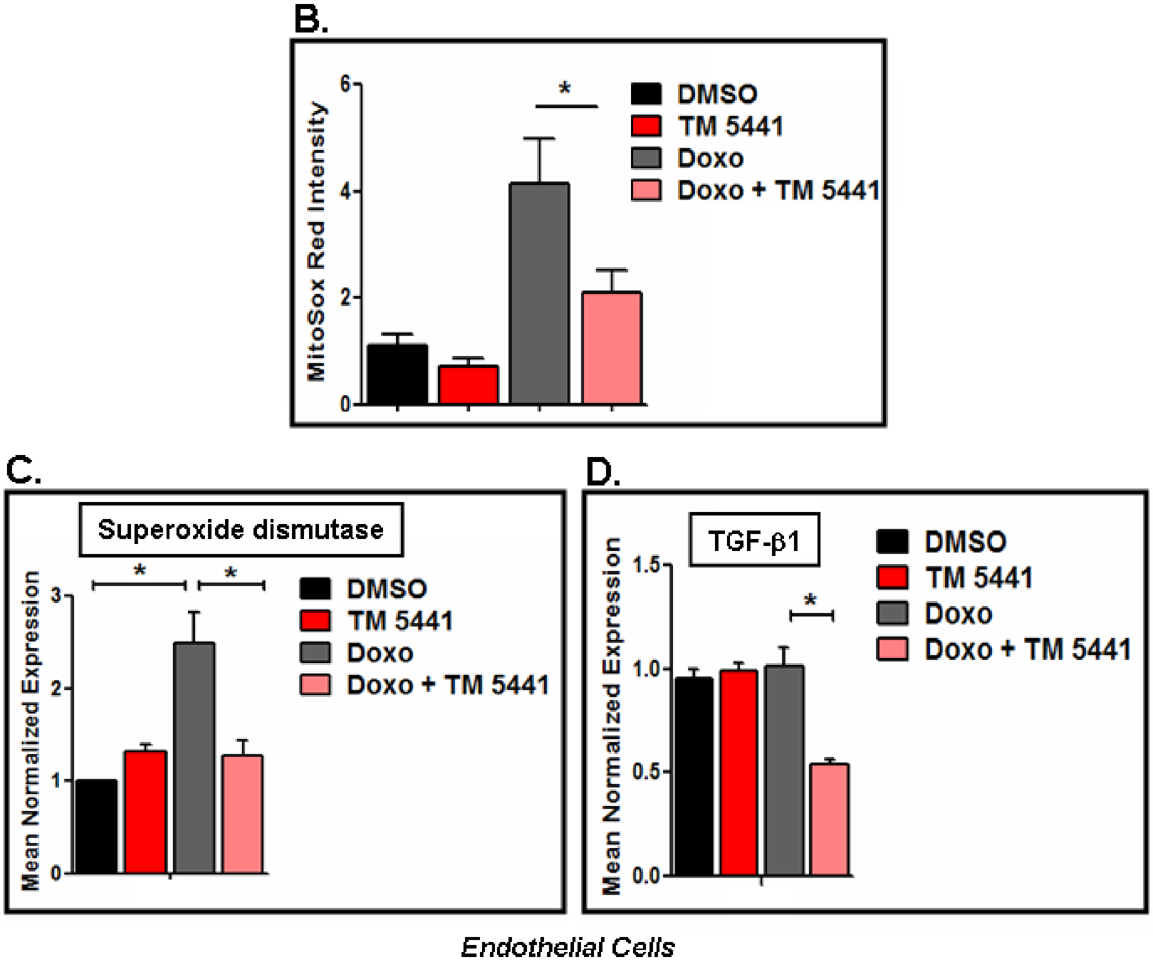

Supplemental Figure 4. PAI-1 inhibitor TM5441 inhibits Doxorubicin-induced ROS generation in endothelial cells. EA.hy926 endothelial cells were pretreated with TM5441 in triplicate for 24 h followed by Doxorubicin (Doxo) treatment for 2 h. The representative images are shown. The levels of oxidized superoxide positive cells were measured **(A,B)**. * denotes $p<0.05$ (**B**). Batches of endothelial cells were pretreated with TM5441 in triplicate followed by Doxorubicin treatment for 4 days. Total RNA isolated from control and treated cells. The levels of ROS quenching factor and ROS target genes were measured by qPCR using gene specific primers (**C,D**). * denotes $p=0.017$ (**C**) and $p=0.011$ (**D**).
